# Assessment of perception, attitude, and practice of primary care practitioners towards allergic rhinitis practice guidelines: Development and validation of a new questionnaire

**DOI:** 10.1016/j.waojou.2020.100482

**Published:** 2020-11-18

**Authors:** Baharudin Abdullah, Ramaprabah Kandiah, Nik Fariza Husna Nik Hassan, Ahmad Filza Ismail, Zahiruddin Wan Mohammad, De Yun Wang

**Affiliations:** a Department of Otorhinolaryngology- Head & Neck Surgery, School of Medical Sciences, Universiti Sains Malaysia Health Campus, 16150 Kubang Kerian, Kelantan, Malaysia; b Department of Community Medicine, School of Medical Sciences, Universiti Sains Malaysia Health Campus, 16150 Kubang Kerian, Kelantan, Malaysia; c Department of Otolaryngology, Yong Loo Lin School of Medicine, National University of Singapore, Singapore, 119228, Singapore

**Keywords:** Primary care practitioners, Allergic rhinitis guidelines, Perception, Attitude, Practice, IgE, immunoglobulin, EAR, Allergic rhinitis, PCPs, Primary care practitioners, ORL, Otorhinolaryngologist, ARIA, Allergic Rhinitis and its Impact on Asthma, GINA, Global Initiative for Asthma, CVI, Content Validity Index, S-CVI/Ave, Scale-level CVI of averaging calculation method, FVI, Face Validity Index, 2-PL IRT, Two-parameter logistic item response theory, KMO, Kaiser-Meyer-Olkin test, EFA, exploratory factor analysis

## Abstract

**Background:**

Primary care practitioners (PCPs), being the front liners, play an important role in treating allergic rhinitis (AR). As there is no proper tool to assess their perception, attitude, and practice in utilizing the guidelines, we aimed to develop and validate a new questionnaire for such purpose.

**Methods:**

The development phase consists of both literature and expert panel review. The validation phase consists of content validity, face validity, and construct validity. Cronbach's alpha was used to verify internal consistency. The development phase produced a questionnaire with 3 domains: perception, attitude, and practice consisting of 60 items (PAP-PCP questionnaire). Item response theory analysis for perception demonstrated the difficulty and discrimination values were acceptable except for 3 items. Exploratory factor analysis for attitude and practice domains showed the psychometric properties were good except for 3 items in practice domain. Experts judgement was used to decide on the final selection of questionnaire which consists of 59 items.

**Results:**

The final validated questionnaire has 3 domains with 59 items. All domains had Cronbach's alpha above 0.65 which was reliable. 302 physicians completed the questionnaire. 98% PCPs diagnosed AR based on clinical history. Although, majority agree AR guidelines is useful (67%), they had difficulty in using it to classify AR (54.9%) and determine AR severity (73.9%). Oral anti-histamines (first and second generation) were the most prescribed (>75%) followed by intranasal corticosteroids (59%) and combined intranasal corticosteroid and oral anti-histamine (51%). Majority agreed that treatment efficacy (81.8%), adverse effects (83.8%), fear of adverse effects (73.5%), route of administration (69.4%), dosing frequency (72.5%), taste (64.6%) and cost (73.5%) affect treatment compliance.

**Conclusions:**

The newly developed and validated questionnaire is a promising instrument in understanding the treatment gap in AR. Although further testing and refinement are needed, it provides an initial means for evaluating knowledge and understanding of PCPs in treating AR.

## Introduction

Allergic rhinitis (AR) is an inflammatory disease of the nasal mucous membranes. An allergen exposure of allergic individuals results in an immunoglobulin E (IgE)-mediated inflammatory response, which can be manifested clinically as nasal congestion, postnasal drainage, rhinorrhea, nasal itching, sneezing, and itchy or watery eyes.^1^^,^^2^ For patients suffering from AR, primary care practitioners (PCPs) are often their first source of medical advice.^3^ It is one of the top-ten reasons for a visit to primary care clinics, and AR was estimated to be 10–40% of the total patient visits in approximately 50% of primary care clinics.^4^^,^^5^ It has been reported in a population-based survey, that 71% of patients suffering from rhinitis visited a primary care physician and only 18% consulted an otorhinolaryngology (ORL) specialist.^5^ This emphasizes the key role of PCPs in the diagnosis, treatment, and follow-up of AR.^6^

As many patients rely on their PCPs for diagnosis and treatment, primary care practice represents a crucial and important profession to be evaluated as part of the management strategy of AR.^3^ The Allergic Rhinitis and its Impact on Asthma (ARIA) guidelines, is the first evidence-based guideline for AR and has been published and updated regularly since 2001, with the aim to improve the care of AR patients.^7^ Among the recommendations from the guidelines are allergen avoidance, pharmacotherapy, and allergen immunotherapy. Despite the availability of multiple treatment options as mentioned in the guidelines, AR continues to be treated sub-optimally. There are indications that ARIA guidelines have not been followed and probably neglected by PCPs. A study among Italian general practitioners showed that they treat patients independently of guidelines.^8^ In Canada, unprompted awareness of ARIA guidelines was nonexistent among primary care practice (0%).^1^ This is partly due to the use of local guidelines in their practices instead of ARIA. Nonetheless, there is very limited information available on the reasons why the ARIA guidelines have not been properly utilized despite being widely accessible. One of the reasons could be that there is a lack of specific instrument to evaluate the knowledge and understanding of primary care practitioners (PCPs) with regard to ARIA guidelines. Thus, we aimed to develop and validate a questionnaire (PAP-PCP) to assess their perception, attitude, and practice towards ARIA guidelines. The PAP-PCP questionnaire (Appendix A) is defined as an instrument for the baseline assessment of perception, attitude, and practice of primary care practitioners towards AR practice guidelines.

## Methodology

### Study design and regulatory approvals

The study was a non-interventional prospective observational study. Ethical approval was obtained from the local human ethics committees. Written consent in English was obtained from each participant prior to conducting the survey.

### Study population

This study was conducted amongst PCPs in the out-patient setting of hospitals, health clinics, and private clinics from April 19, 2018 until December 18, 2018. Sample size was determined using the factor analysis method^9^ with a subject to variable ratio of 1:5, and it showed 300 participants were required. Purposive sampling method was used for recruitment. Inclusion criteria were PCPs working as general practitioners at their private clinics or medical officers at hospital outpatient clinics or health clinics and able to understand English. The PCPs in the vicinity were identified and informed about the study. The questionnaire was hand-delivered to those who were willing to take part and hand-collected once they had completed the questionnaire. There were 3 phases in this study. The first phase was development, the second phase was validation, and the third phase was reliability of the questionnaire.

### Development of questionnaire

In the first phase, the PAP-PCP questionnaire, a self-administered questionnaire was developed from consultation among experts and adaptation from literature reviews.^10^, ^11^, ^12^ The experts consisted of otorhinolaryngologists and public health physicians. The concepts identified in the literature review were used in the selection of items and formation of the relevant PAP-PCP questionnaire sections in the questionnaire. The newly developed questionnaire had 2 parts. The first part was demographics which included age, gender, ethnicity, years of practice, main workplace, and the total number of patients seen in a week (number of patients with rhinitis or asthma or both seen per week) (Table 1). The second part had 3 constructs (perception, attitude, and practice) with a total of 60 items (Table 1). In the perception domain, there were 9 items and the dichotomous scale response to each item (expressed as "yes, not sure, and no"). The attitude domain has 28 items and the likert scale response to each item (expressed as "strongly agree, agree, neutral, disagree, and strongly disagree"). The practice domain has 23 items and the likert scale response to each item (expressed as "always, often, sometimes, seldom, and never").

**Table 1:** Questionnaire components included in the Perception, Attitude and Practice of Primary Care Practitioners (PAP-PCP) Questionnaires

Sections	No. of Items	Concepts measured	Response options
Perception (part 2)	9	Existence of AR practice guidelines, types, category and severity of AR	yes, not sure, no
Attitude (part 2)	28	General attitude, behaviour and cognitive factors with regard to diagnosing, classifying and treating AR	strongly agree, agree, neutral, disagree, strongly disagree
Practice (part 2)	23	Common practice for diagnosing AR, preferred practice of allergic testing and treatment options	always, often, sometimes, seldom, never

Perception is defined as the way in which something is regarded, understood, or interpreted. In short, it is the result of a cognitive process. The perception questions explored concepts of existence of AR practice guidelines, diagnosing AR, common symptoms of AR, and classification and severity of AR (Table 1). An attitude is a settled way of thinking or feeling about something. The attitude questions were designed to assess PCPs' general attitudes, behaviours and cognitive factors with regard to diagnosing, classifying, and treating AR (Table 1). Practice is the actual application of an idea, belief, or method. The practice questions evaluated PCPs' common practice for diagnosing AR and preferred practice of allergic testing and treatment (Table 1).

### Validation and reliability of questionnaire

In the second phase, the validation of PAP-PCP questionnaire was done based on 3 levels of evidence which are content validity, face validity, and construct validity. The Content Validity Index (CVI) assessed relevance and representability of each item to a specific domain by the panel of experts.^13^ Nine experts and researchers in the field (7 otorhinolaryngologists and 2 public health physicians) were chosen for this task. The otorhinolaryngologists served as the subject matter experts, and the public health physicians were the questionnaire design experts. The expert groups pretested the questionnaire to evaluate for potential problems when used by respondents. Each reviewer independently rated the relevance of each item for each domain of the questionnaire to the conceptual framework using a 4-point Likert scale (1 = not relevant, 2 = somewhat relevant, 3 = relevant, 4 = very relevant). For example, if 5 of 8 content experts rate an item as relevant (3 or 4) the CVI would be 5/8 = 0.62, which does not meet the 0.87 (7/8) level required and implies that the item should be dropped. Another parameter was Scale-level CVI of averaging calculation method (S-CVI/Ave). S-CVI/Ave must be 0.83 and above to be considered as an acceptable content validity. All items were valid with CVI ranging from 0.88 to 1.00, S-CVI/Ave ranging from 0.94 to 1.00 and they were retained as per the initial questionnaire. Face Validity Index (FVI) assessed comprehensibility and clarity of each item by respondents. Ten PCPs not involved in the study proper, took part in this assessment. The items were rated based on Likert scale ranging from 1 (not clear or not comprehensible) to 4 (very clear or very comprehensible). Ninety percent indicated they understood the questions and found them easy to answer, and 90% indicated the appearance and layout would be acceptable to the intended target group. The final draft of the questionnaire at this stage contained two parts. Part 1 is the demography and part 2 contains the questionnaire with 3 domains (perception, attitude and practice) consisting of 60 items.

The perception domain was analysed by two-parameter logistic item response theory (2-PL IRT) analysis, using R software (R version 3.6.1 for Windows, © the R foundation), as it consists of unidimensional items with dichotomous responses. Difficulty in the range of −3 to +3 and discrimination in the range of 0.35–2.5 were considered acceptable.^14^^,^^15^

The exploratory factor analysis and Cronbach's alpha were used to measure construct validity and internal consistency of the remaining 2 domains in the questionnaire (attitude and practice).^14^ The factor analysis, Kaiser-Meyer-Olkin test (KMO) and Bartlett's test of sphericity were computed to identify the items to be included in the final analysis. A typical factor analysis was performed based on Pearson correlations since the Likert scale could be treated as an interval or ratio scale. Factor loadings >0.3 was considered acceptable.^16^ Once the validity procedures were completed, the final version of the questionnaire was examined to assess its reliability which was the third phase. The internal consistency reliability, a Cronbach's alpha coefficient >0.65 was considered acceptable.^16^

### Statistical analysis

The data were tabulated using Microsoft Excel and analysed using the Statistical Package for Social Sciences, SPSS 24 for Windows. Descriptive statistics was used to summarise the socio-demographic characteristics of subjects. Numerical data was represented as mean (SD) and frequency (n-%). Questions were coded based on a Likert-scale, on which a more desirable answer was given a higher score than a less desirable answer. Factor loadings were used to extract factors and items in exploratory factor analysis (EFA). Cronbach's alpha coefficient was used as an estimate of the internal consistency of the questionnaire.

## Results

### Demography

A total of 302 PCPs participated in this study consisting of 118 men and 184 women. The age ranged from 28 to 73 years old with mean age of 36.4. The majority of them were Malays 271 (89.7%), followed by Chinese 23 (7.6%), Indian 7 (2.3%), and others 1 (0.3%) (Table 2). The mean years of practising were 10.0 years and the mean estimate of total number of patients seen per week were 153.5, with mean of 10 rhinitis patients, 10 asthma patients, and 5 both rhinitis and asthmatic patients seen per week. Their responses towards the PAP-PCP questionnaire are shown in Table 3, Table 4 and Table 5.

**Table 2:** Characteristics of the study respondents.

Variable	Mean (SD)	n (%)
Age of respondent	36.42 (10.15)	
Gender		
Male		118 (39.1)
Female		184 (60.9)
Race		
Malay		271 (89.7)
Chinese		23 (7.6)
Indian		7 (2.3)
Other		1 (0.3)
Year of Practising	10.07 (8.57)	
Estimate patient seen per week	153.52 (101.52)	
No of patient rhinitis seen per week	10 (15)^a^	
No of patient asthma seen per week	10 (15)^a^	
No of patient having both rhinitis and asthma seen per week	5 (9)^a^	

a Median (interquartile range)

**Table 3:** Perception towards the ARIA guidelines

Question	Response n (%)			a Cronbach's alpha
	Yes	Not sure	No	
**P-Q1.** Do you know allergic rhinitis and its impact on asthma (ARIA) guidelines?	201 (66.6)	52 (17.2)	49 (16.2)	0.7
**P-Q2.** Do you know global initiative for asthma (GINA) guidelines?	273 (90.4)	15 (5)	14 (4.6)	0.7
**P-Q3.** Do you know other guidelines for allergic rhinitis?	43 (14.2)	117 (38.7)	142 (47.0)	0.7
**P-Q4.** Do you know rhinitis can be divided into AR and non-AR?	194 (64.2)	54 (17.9)	54 (17.9)	0.7
**P-Q5.** Is an evaluation of asthma necessary for AR patients?	251 (77.5)	41 (13.6)	10 (3.3)	0.7
**P-Q6.** Do you know how to diagnose AR?	234 (77.5)	56 (18.5)	12 (4)	0.7
**P-Q7.** Do you know the common symptoms of AR?	296 (98)	4 (1.3)	2 (0.7)	0.7
**P-Q8.** Do you know how to classify allergic rhinitis?	136 (45)	104 (34.4)	62 (20.5)	0.7
**P-Q9.** Do you know the severity of allergic rhinitis?	80 (26.5)	148 (49)	74 (24.5)	0.7

a Cronbach's alpha coefficient > 0.65 was considered acceptable

**Table 4:** Attitude towards the ARIA guidelines

Question	Response n (%)					a Cronbach's alpha
	Strongly Agree	Agree	Neutral	Disagree	Strongly Disagree	
**A-Q10.** ARIA guidelines is useful in categorizing patients?	74 (24.5)	148 (49)	80 (26.5)	0 (0)	0 (0)	0.83
**A-Q11.** A new subdivision of allergic rhinitis has been proposed as “intermittent” and “persistent"	62 (20.5)	137 (45.4)	102 (33.8)	1 (0.3)	0 (0)	0.83
**A-Q12.** The severity of allergic rhinitis has been classified as “mild” or “moderate/severe” depending on the severity of the symptom and quality of life outcomes?	97 (32.1)	164 (54.3)	41 (13.6)	0 (0)	0 (0)	0.83
**A-Q13.** The diagnosis of allergic rhinitis is based upon the concordance between a typical history of allergic symptoms and allergy test?	39 (12.9)	145 (48)	75 (24.8)	36 (11.9)	7 (2.3)	0.78
**A-Q14.** ARIA guidelines is useful for the treatment of your allergic rhinitis patients?	95 (31.5)	143 (47.4)	60 (19.9)	4 (1.3)	0 (0)	0.83
**A-Q15.** I feel this medication is effective in treating AR patients						
a) First generation oral anti-histamines	53 (17.5)	165 (54.6)	69 (22.8)	13 (4.3)	2 (0.7)	0.83
b) Second generation oral anti-histamines	77 (25.5)	176 (58.3)	46 (15.2)	2 (0.7)	1 (0.3)	0.83
c) intranasal corticosteroids	115 (38.1)	148 (49)	39 (12.9)	0 (0)	0 (0)	0.83
d) oral antihistamines and decongestants	77 (25.5)	181 (59.9)	41 (13.6)	3 (1.0)	0 (0)	0.83
e) leukotriene antagonist	29 (9.6)	122 (40.4)	126 (41.7)	19 (6.3)	6 (2)	0.78
f) oral corticosteroids	23 (7.6)	83 (27.5)	112 (37.1)	64 (21.2)	20 (6.6)	0.78
g) immunotherapy	16 (5.3)	60 (19.9)	154 (51)	51 (16.9)	21 (7)	0.78
h) intranasal antihistamine	30 (9.9)	105 (34.8)	129 (42.7)	31 (10.3)	7 (2.3)	0.78
**A-Q16.** I feel this medication is safe in treating AR patients-						
a) First generation oral anti-histamines	98 (32.5)	158 (52.3)	40 (13.2)	5 (1.7)	1 (0.3)	0.83
b) Second generation oral anti-histamines	112 (37.1)	166 (55)	24 (7.9)	0 (0)	0 (0)	0.83
c) intranasal corticosteroids	90 (29.8)	151 (50)	57 (18.9)	4 (1.3)	0 (0)	0.83
d) oral antihistamines and decongestants	65 (21.5)	179 (59.3)	54 (17.9)	4 (1.3)	0 (0)	0.83
e) leukotriene antagonist	34 (11.3)	112 (37.1)	141 (46.7)	9 (3)	6 (2)	0.83
f) oral corticosteroids	11 (3.6)	80 (26.5)	125 (41.4)	61 (20.2)	25 (8.3)	0.78
g) immunotherapy	7 (2.3)	57 (18.9)	167 (55.3)	55 (18.2)	16 (5.3)	0.78
h) intranasal antihistamine	29 (9.6)	107 (35.4)	140 (46.4)	20 (6.6)	6 (2)	0.78
**A-Q17.** I feel that the treatment compliance is affected by these factors						
a) adverse effects produced by medications	95 (31.5)	157 (52)	43 (14.2)	7 (2.3)	0 (0)	0.87
b) fears of adverse effects reported	65 (21.5)	157 (52)	67 (22.2)	11 (3.6)	2 (0.7)	0.87
c) route of administration	69 (22.8)	140 (46.4)	70 (23.2)	23 (7.6)	0 (0)	0.87
d) frequency of doses	84 (27.8)	135 (44.7)	53 (17.5)	29 (9.6)	1 (0.3)	0.87
e) efficacy of on-going treatment	89 (29.5)	158 (52.3)	44 (14.6)	11 (3.6)	0 (0)	0.87
f) cost of medication	86 (28.5)	136 (45)	65 (21.5)	10 (3.3)	5 (1.7	0.87
g) taste	63 (20.9)	132 (43.7)	73 (24.2)	25 (8.3)	9 (3)	0.87

a Cronbach's alpha coefficient > 0.65 was considered acceptable

**Table 5:** Practice according to the ARIA guidelines

Question	Response n (%)					aCronbach's alpha
	Always	Often	Sometimes	Seldom	Never	
**PR-Q18.** I diagnose my patient with AR by						
a) clinical history^b^	194 (64.2)	102 (33.8)	6 (2)	0 (0)	0 (0)	–
b) anterior rhinoscopy	46 (15.2)	52 (17.2)	66 (21.9)	36 (11.9)	102 (33.8)	0.66
c) allergy testing	9 (3)	17 (5.6)	45 (14.9)	39 (12.9)	192 (63.6)	0.90
d) imaging paranasal sinuses	3 (1)	18 (6)	46 (15.2)	46 (15.2)	189 (62.6)	0.90
e) nasal endoscopy	7 (2.3)	18 (6)	35 (11.6)	33 (10.9)	209 (69.2)	0.90
**PR-Q19.** In allergy testing,I use						
a) skin prick test	25 (8.3)	41 (13.6)	39 (12.9)	14 (4.6)	183 (60.6)	0.90
b) skin patch test	16 (5.3)	30 (9.9)	48 (15.9)	20 (6.6)	188 (62.3)	0.90
c) serum total IgE	17 (5.6)	25 (8.3)	42 (13.9)	19 (6.3)	199 (65.9)	0.90
d) serum specific IgE	14 (4.6)	20 (6.6)	41 (13.6)	20 (6.6)	207 (68.5)	0.90
e) serum eosinophilia	13 (4.3)	37 (12.3)	42 (13.9)	21 (7)	189 (62.6)	0.90
f) none of the above^c^	123 (40.7)	13 (4.3)	41 (13.6)	23 (7.6)	102 (33.8)	–
**PR-Q20.** I treat AR patient with						
a) First generation oral anti-histamines^d^	106 (35.1)	131 (43.4)	40 (13.2)	20 (6.6)	5 (1.7)	–
b) Second generation oral anti-histamine	132 (43.7)	127 (42.1)	28 (9.3)	4 (1.3)	11 (3.6)	0.66
c) intranasal corticosteroids	66 (21.9)	113 (37.4)	58 (19.5)	28 (6.3)	36 (11.9)	0.66
d) oral antihistamines and decongestants	61 (20.2)	144 (47.7)	61 (5.3)	16 (5.3)	20 (6.6)	0.86
e) leukotriene antagonist	10 (3.3)	39 (12.9)	80 (26.5)	55 (18.2)	118 (39.1)	0.86
f) intranasal decongestants	19 (6.3)	67 (22.2)	81 (26.8)	49 (16.2)	86 (28.5)	0.86
g) oral corticosteroids	13 (4.3)	51 (16.9)	75 (24.8)	61 (20.2)	102 (33.8)	0.86
h) immunotherapy	0 (0)	8 (2.6)	43 (14.2)	41 (13.6)	210 (69.5)	0.86
i) intranasal antihistamine	8 (2.6)	34 (11.3)	55 (18.2)	48 (15.9)	157 (52)	0.86
j) combination of antihistamines and intranasal steroids	58 (19.2)	98 (32.5)	61 (20.2)	28 (9.3)	57 (18.9)	0.66
k) combination of antihistamines and leukotriene antagonist	9 (3.0)	35 (11.6)	74 (24.5)	53 (17.5)	131 (43.4)	0.86
l) combination of leukotriene antagonist and intranasal steroids	8 (2.6)	34 (11.3)	66 (21.9)	50 (16.6)	144 (47.7)	0.86

a Cronbach's alpha coefficient > 0.65 was considered acceptable

b PR-Q18a had low factor loading but following expert judgement was retained

c PR-Q19f had low factor loading and following expert judgement was removed

d PR-Q 20a had low factor loading but following expert judgement was retained

### Validity and reliability of the questionnaire

a. Perception domain

Based on the two-parameter logistic item response theory (2PL-IRT) analysis, the psychometric properties of the perception domain were good. For the difficulty parameter, all the perception items were within acceptable range of −3 to +3. In terms of discrimination, most of the items were within acceptable range (0.35–2.5), except for question P-Q7 (**Do you know the common symptoms of AR?**), P-Q8 (**Do you know how to classify allergic rhinitis?**) and P-Q9 (**Do you know the severity of allergic rhinitis?**) which were out of the acceptable range (>2.5). However, following expert consensus and judgement, all questions were retained because the contents of the items were deemed important and they had acceptable difficulty values with a reliable Cronbach's alpha (0.7).

b. Attitude domain

Kaiser-Meyer-Olkin Measure of Sampling Adequacy for attitude domain was 0.77 (adequate) and Barlett's test of the sphericity was significant (p value < 0.001). Three factors were extracted in attitude domain, which were factor A1 with 13 items (A-Q10, A-Q11, A-Q12, A-Q14, A-Q15a, A-Q15b, A-Q15c, A-Q15d, A-Q16a, A-Q16b, A-Q16c, A-Q16d and A-Q16e), factor A2 with 8 items (A-Q13, A-Q15e, A-Q15f, A-Q15g, A-Q15h, A-Q16f, A-Q16g and A-Q16h) and factor A3 with 7 items (A-Q17a, A-Q17b, A-Q17c, A-Q17d, A-Q17e, A-Q17f and A-Q17g). All items were retained as their factor loadings were acceptable (>0.3). Reliability for each factor in attitude domain were above 0.65. Reliability of factor A1 was 0.83, factor A2 was 0.78 and factor A3 was 0.87 (Table 4).

c. Practice domain

**Fig. 1:**
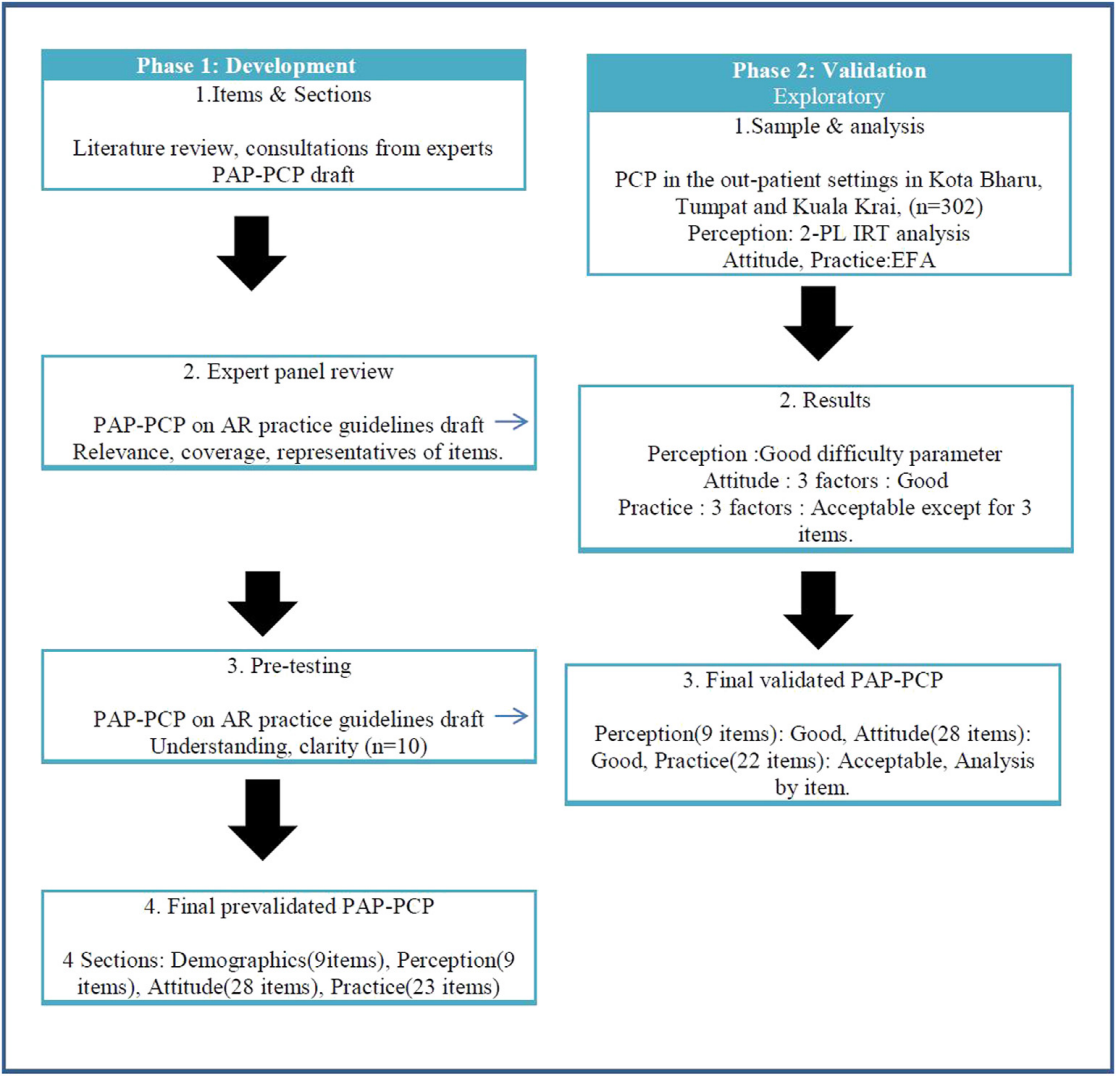
A summary of the development and validation stages of the questionnaires

For the practice domain, Kaiser-Meyer-Olkin Measure of Sampling Adequacy was 0.89 (adequate) and Barlett's test of the sphericity was significant (p value < 0.001). Similarly, 3 domains were extracted; factor PR 1 with 8 items (PR-Q18c, PR-Q18d, PR-Q18e, PR-Q19a, PR-Q19b, PR-Q19c, PR-Q19d, PR-Q19e), factor PR 2 with 8 items (PR-Q20d, PR-Q20e, PR-Q20f, PR-Q20g, PR-Q20h, PR-Q20i, PR-Q20k, PR-Q20l) and factor PR 3 with 4 items (PR-Q18b, PR-Q20b, PR-Q20c, PR-Q20j). All items in the 3 domains had acceptable factor loadings (>0.3) except for 3 items. Three items, PR-Q18a (**I diagnose my patients with AR by clinical history**), PR-Q19f (**In allergy testing, I use none of the above**) and PR-Q20a (**I treat AR patients with 1**st **generation oral anti-histamine**) had low factor loadings. Following experts consensus and judgement, 2 items PR-Q18a and PR-Q20a were retained while item PR-Q19f was removed. Reliability for each factor in practice domain were 0.90, 0.86, and 0.66 respectively (Table 5). The final validated questionnaires have 3 domains with 59 items. A summary of the development and validation stages of the questionnaire is presented in Fig. 1.

### Assessment of the validated items

For the perception domain, PCPs had higher frequencies of correct answers in 6 items. However, there were 3 items (**P-Q3, P-Q8, P-Q9)** which had lower scores. More than 50% of PCPs answered incorrectly for those items and these findings suggest a knowledge gap amongst PCPs. PCPs recognised ARIA and Global Initiative for Asthma (GINA) guidelines, able to differentiate AR from non-AR, aware of the role of appraising and diagnosing asthma, recognised common symptoms of AR, not aware of other AR practice guidelines, and had difficulties in classifying and determining the severity of AR (Table 3). For the attitude domain, a high percentage of PCPs, more than 65% responded favourably to the importance of ARIA guidelines in diagnosing, categorizing, and determining the severity of AR. In addition, the PCPs seemed to be ignorant and unaware of the benefits of newer antihistamine drugs and treatment like the immunotherapy (Table 4). For the practice domain, 98% PCPs diagnosed AR based on clinical history. Less than 10% of them used modalities like imaging, nasal endoscopy, and allergic testing to facilitate in the diagnosis of AR. In terms of allergic testing, PCPs' favourite practice was the skin prick test (21.9%), followed by blood investigations such as serum eosinophilia, serum total IgE and serum specific IgE (16.6%, 13.9% and 11.2% respectively). The response for treatment choice in the practice domain revealed that majority of PCPs preferred prescribing oral medications like anti-histamine (first and second generation), accounting for more than 75% compared to intranasal corticosteroids which was only 59% and combination of intranasal corticosteroid and oral anti-histamine was about 51% (Table 5).

For physicians who did not find the guidelines useful, their patients were treated with oral antihistamine (first and second generation) (43%), combined oral antihistamine and oral decongestant (20%), intranasal corticosteroid as a monotherapy (17%), and combined intranasal corticosteroid and oral antihistamine (12%). For physicians who did not find immunotherapy safe, they prescribed oral antihistamine (first and second generation) (45%), combined oral antihistamine and decongestant (21%), intranasal corticosteroid as a monotherapy (18%), and combined oral antihistamine and intranasal corticosteroid (17%). For physicians who never used allergy tests, they prescribed oral antihistamine (first and second generation) (47%), combined oral histamine and decongestant (20%), and combined oral antihistamine and oral decongestant (18%).

## Discussion

AR with its increasing prevalence, represents a major global health concern. As the front liners in managing AR patients, PCPs play an important role in providing the appropriate care. There are evidences that unmet needs of patients have not been addressed despite AR guidelines such as ARIA have been revised and disseminated to health care workers worldwide.^10^^,^^17^^,^^18^ There are no proper studies being done among PCPs to assess their knowledge and understanding of AR guidelines partly because there is no standardized tool available. Most studies developed their own questionnaires for assessment and since there is no conformity in the questionnaires used, consistent and uniform interpretations of their results are difficult to assimilate. It is important for us to have a validated and reliable questionnaire to gauge the perception, attitude, and practice of PCPs towards AR practice guidelines. The availability of such instrument is critical to identify the knowledge gap and ensure proper measures can be taken. In this study, the PAP-PCP questionnaire was developed and validated to serve as an essential starting point for further evaluation using larger sample size to determine the reason of poor recognition of ARIA guidelines leading to suboptimal treatment of AR.

### Development and evaluation of the questionnaire

The initial questionnaire following the content and face validation, had 3 domains (perception, attitude, and practice) with 60 items. Overall, the perception domain showed good psychometric properties based on the difficulty and discriminatory parameters of the items. The discrimination parameters were mostly within the acceptable range except for items P-Q7 (**Do you know the common symptoms of AR?**), P-Q8 (**Do you know how to classify AR**) and P-Q9 (**Do you know the severity of allergic rhinitis?**). However, based on the content validity, the 3 items were found to be relevant and adequately represent the domain. Content validity is a prerequisite for any other forms of validity; thus, they should be given the highest priority in the development of any new inventory.^16^ Therefore, all 3 items were retained, since these questions were essential to assess the AR practice guidelines such as ARIA and the experts have deemed, they provide strong evidence for the content validity. Three items had low factor loadings (<0.3) in the practice domain, in which 2 items were retained and 1 was removed. Item PR-Q18a (**I diagnose my patients with AR by clinical history**) was retained as clinical history is the best way of diagnosing AR by the PCPs while item PR-Q20a (**I treat AR patients with 1**st **generation oral anti-histamine**) was retained, because it is the first line treatment for mild and moderate AR according to ARIA guidelines. Item PR-Q19f (**In allergy testing, I use none of the above**) was removed as the experts felt that the item did not fit that domain (under presented construct).

### Assessment of the perception, attitude and practice

For the perception domain (**P-Q1, P-Q2**), the results suggested that PCPs knew GINA better than ARIA guidelines. This probably reflects their concerns about asthma as most perceived asthma as a life-threatening condition and should be properly treated. They knew that asthma must be evaluated in AR (**P-Q5**) but surprisingly despite knowing the AR symptoms (**P-Q7**), the PCPs have low awareness of AR classification (**P-Q8**) and the severity of the disease (**P-Q9**). On the other hand, the results for the attitude domain, suggested that more than half of PCPs agree that ARIA is useful in categorizing AR patients and acknowledge classifying patients based on severity (**A-Q10, A-Q11, A-Q12, A-Q14**). The discord between perception and attitude reflect the knowledge gap among PCPs in the significance and proper use of ARIA guidelines to classify AR and determine AR severity for proper treatment selection in their practice. The other interesting finding is despite only 201 respondents knew about ARIA guidelines, but 222 respondents agreed or strongly agreed that ARIA was useful for categorizing patients. We believe this is due to social desirability bias. The respondents might be pressured to answer that they agree about the usefulness of the guidelines to avoid being perceived as ignorant even though they have been reassured that their answers would be confidential. Majority of the PCPs (67.2%) agreed that the diagnosis of AR patients should be based on clinical history and allergic testing (**A-Q13**). When assessed for their attitude towards AR treatment (**A-Q15, A-Q16**), most PCPs agreed that oral anti-histamines, both first generation (72.1%) and second generation (83.8%), oral antihistamines and decongestants (85.4%), leukotriene antagonists (50%), and intranasal corticosteroids (87.1%) were effective and safe in treating AR patients. This may explain their preference (**PR-Q20**) of prescribing oral anti-histamines, first generation (78.5%) or second generation (85.8%), oral antihistamines and decongestants (67.9%), intranasal corticosteroids (59.3%), and oral antihistamines and intranasal corticosteroids (51.7%). The choice of medications in their practice reflects their consideration for patient's treatment compliance (**A-Q17**). Most PCPs agreed that treatment efficacy (81.8%), adverse effects (83.8%), fear of adverse effects (73.5%), route of administration (69.4%), dosing frequency (72.5%), taste (64.6%), and cost (73.5%) affect treatment compliance. Those factors might explain their choice of first line therapy using oral antihistamines for all AR patients to be followed by combination therapy only when patients do not response to first line treatment despite ARIA recommending treatment based on the severity of AR. The use of single modality in AR treatment also could be due to the cost factor as most patients attending PCPs clinic are cash paying and PCPs need to ensure their treatment is affordable. The overall results indicate that there was a knowledge gap among PCPs which require more educational program among PCPs to create better recognition and understanding in better utilization of ARIA guidelines.

### Limitations

We acknowledged several limitations of the present study. English is not the primary language in our country and there might be concern in the respondent's ability to answer the questionnaire appropriately with the possibility that the results might be influenced by this limitation. However, English is widely used and taught in schools and universities. In fact, for the medical program, English medium is used throughout the teaching sessions and professional examination. Additionally, English language is used widely among the medical fraternity for communication. Furthermore, as all participants selected in the present study were based on their competency in English language, we believe the results are valid and appropriate.

Most of the participants involved in the present study were from a younger age group (mean age 36.4) and the results might be different with the involvement of older age group. Hence, we recommend more studies to validate the questionnaire in larger representative samples to ascertain its generalizability.

## Conclusions

The newly developed and validated questionnaire is a promising instrument for the evaluation of the perception, attitude, and practice of PCPs towards AR. This is an important step towards understanding the treatment gap in AR. Although further testing and refinement are needed, it provides an initial means for evaluating the knowledge and understanding of PCPs towards ARIA guidelines.

## Funding

None.

## Authors’ contributions

BA, RK, NFHNH, AFI, ZWM, DYW participated in the design of the study, drafted the manuscript, collected the data, analyze the data and revised the manuscript for critical content. All authors read and approved the final manuscript.

## Ethics approval

Ethical approval was obtained from Human Research Ethics Committee USM (USM/JEPeM/17110581) and Medical Research & Ethics Committee (NMRR-17-3342-38820). Written consent in English was obtained from each participant prior to conducting the survey.

## Consent for publication

All authors have approved the manuscript for submission.

## Availability of data and materials

The datasets used and/or analysed during the current study are available from the corresponding author on reasonable request.

## Declaration of competing interest

The authors declare that they have no competing interests.
