# Supplementary material for: Assessment of perception, attitude, and practice of primary care practitioners towards allergic rhinitis practice guidelines: Development and validation of a new questionnaire
Source: World Allergy Organ J. 2020 Nov 18;13(12):100482. doi: 10.1016/j.waojou.2020.100482 (PMC7689322; doi:10.1016/j.waojou.2020.100482)
Supplement: Multimedia component 1 [file mmc1.docx]

**Appendix A.** Perception, attitude and practice of primary care practitioners (PAP-PCP) towards allergic rhinitis practice guidelines questionnaire.

| **Part I.** Demographics.  Fill in the appropriate information that best represents you.  Study ID: _________ | |
| --- | --- |
| 1. | 1. Age : ______ |
| 2. | 1. Gender : Male ____ Female ____ |
| 3. | 1. Ethnicity :___________ |
| 4. | 1. Years of practicing:___________ |
| 5. | 1. Main workplace:________ |
| 6. | Total estimate number of patients seen in a week time: _________________ |
| 7. | The number of patients with rhinitis seen per week:__________ |
| 8. | The number of patients with asthma seen per week:___________ |
| 9. | The number of patients having both rhinitis and asthma seen per week:___________________ |

**Part 2.** The Perception domain.

| Please tick at the given boxes for each of the statements according to your understanding. | | | |
| --- | --- | --- | --- |
| 1)Do you know allergic rhinitis and its impact on asthma(ARIA) guidelines? | Yes | Not sure | No |
| 2)Do you know global initiative for asthma(GINA) guidelines? |  |  |  |
| 3)Do you know other guidelines for allergic rhinitis(AR)?  If yes state them:____________ |  |  |  |
| 4)Do you know rhinitis can be divided into AR and non-AR? |  |  |  |
| 5)Is an evaluation of asthma necessary for AR patients? |  |  |  |
| 6)Do you know how to diagnose AR? |  |  |  |
| 7)Do you know the common symptoms of AR? |  |  |  |
| 8)Do you know how to classify allergic rhinitis? |  |  |  |
| 9)Do you know the severity of allergic rhinitis? |  |  |  |

**Part 2.** Attitude domain.

| Please use the scale below to describe your agreement and disagreement with each statement.  Please circle at the chosen number.   \| Strongly Agree \| Agree \| Neutral \| Disagree \| Strongly Disagree \| \| --- \| --- \| --- \| --- \| --- \| \| 5 \| 4 \| 3 \| 2 \| 1 \| | | | | | |
| --- | --- | --- | --- | --- | --- | --- | --- | --- | --- | --- | --- | --- | --- | --- | --- |
| 10)ARIA guidelines is useful in categorizing patients? | 5 | 4 | 3 | 2 | 1 |
| 11)A new subdivision of allergic rhinitis has been proposed as "intermittent" and "persistent"? | 5 | 4 | 3 | 2 | 1 |
| 12)The severity of allergic rhinitis has been classified as “mild” or “moderate/severe” depending on the severity of the symptom and quality of life outcomes? | 5 | 4 | 3 | 2 | 1 |
| 13)The diagnosis of allergic rhinitis is based upon the concordance between a typical history of allergic symptoms and allergy tests? | 5 | 4 | 3 | 2 | 1 |
| 14)ARIA guidelines is useful for the treatment of your allergic rhinitis patients? | 5 | 4 | 3 | 2 | 1 |
| 15)I feel this medication is effective in treating AR patients  a)1st generation oral anti-histamines.  b)2nd generation oral anti-histamines.  c)intranasal corticosteroids.  d)oral anti-histamines and decongestants.  e)leukotriene antagonist.  f)oral corticosteroids.  g)immunotherapy.  h)intranasal antihistamine | 5  5  5  5  5  5  5  5 | 4  4  4  4  4  4  4  4 | 3  3  3  3  3  3  3  3 | 2  2  2  2  2  2  2  2 | 1  1  1  1  1  1  1  1 |
| 16)I feel this medication is safe in treating AR patients  a)1st generation oral anti-histamines.  b)2nd generation oral anti-histamines.  c)intranasal corticosteroids.  d)oral anti-histamines and decongestants.  e)leukotriene antagonist.  f)oral corticosteroids.  g)immunotherapy.  h)intranasal antihistamine |  |  |  |  |  |
|  | 5 | 4 | 3 | 2 | 1 |
|  | 5 | 4 | 3 | 2 | 1 |
|  | 5 | 4 | 3 | 2 | 1 |
|  | 5 | 4 | 3 | 2 | 1 |
|  | 5 | 4 | 3 | 2 | 1 |
|  | 5 | 4 | 3 | 2 | 1 |
|  | 5 | 4 | 3 | 2 | 1 |
|  | 5 | 4 | 3 | 2 | 1 |
| 17) I feel that the treatment compliance is affected by these factors  a)adverse effects produced by medications.  b)fears of adverse effects reported.  c)route of administration.  d)frequency of doses.  e)efficacy of ongoing treatment.  f)cost of medication.  g)taste. |  |  |  |  |  |
|  | 5 | 4 | 3 | 2 | 1 |
|  | 5 | 4 | 3 | 2 | 1 |
|  | 5 | 4 | 3 | 2 | 1 |
|  | 5 | 4 | 3 | 2 | 1 |
|  | 5 | 4 | 3 | 2 | 1 |
|  | 5 | 4 | 3 | 2 | 1 |
|  | 5 | 4 | 3 | 2 | 1 |

**Part 2.** Practice domain.

| Please use the scale below to describe the frequency with each statement.  Please circle at the chosen number.   \| Always \| Often \| Sometimes \| Seldom \| Never \| \| --- \| --- \| --- \| --- \| --- \| \| 5 \| 4 \| 3 \| 2 \| 1 \| | | | | | |
| --- | --- | --- | --- | --- | --- | --- | --- | --- | --- | --- | --- | --- | --- | --- | --- |
| 18)I diagnose my patients with AR by    a)Clinical history.  b)Anterior rhinoscopy.  c)Allergy testing.  d)Imaging paranasal sinuses.  e)Nasal endoscopy. | 5 | 4 | 3 | 2 | 1 |
|  | 5 | 4 | 3 | 2 | 1 |
|  | 5 | 4 | 3 | 2 | 1 |
|  | 5 | 4 | 3 | 2 | 1 |
|  | 5 | 4 | 3 | 2 | 1 |
| 19) In allergy testing , I use  a)Skin prick test.  b)Skin patch test.  c)Serum total IgE.  d)Serum specific IgE.  e)Serum eosinophilia.  f)None of the above |  |  |  |  |  |
|  | 5 | 4 | 3 | 2 | 1 |
|  | 5 | 4 | 3 | 2 | 1 |
|  | 5 | 4 | 3 | 2 | 1 |
|  | 5 | 4 | 3 | 2 | 1 |
|  | 5 | 4 | 3 | 2 | 1 |
|  | 5 | 4 | 3 | 2 | 1 |
| 20)I treat AR patients with,  a)1^st^ generation oral anti-histamines.  b)2nd generation oral anti-histamines.  c) intranasal corticosteroids.  d)oral anti-histamines and decongestants.  e)leukotriene antagonists.  f)intranasal decongestants.  g)oral corticosteroids.  h)immunotherapy.  i)intranasal antihistamine.  j)combination of antihistamine and intranasal steroids  k)combination of antihistamine and leukotriene antagonists  l)combination of leukotriene antagonists and intranasal steroids |  |  |  |  |  |
|  | 5 | 4 | 3 | 2 | 1 |
|  | 5 | 4 | 3 | 2 | 1 |
|  | 5 | 4 | 3 | 2 | 1 |
|  | 5 | 4 | 3 | 2 | 1 |
|  | 5 | 4 | 3 | 2 | 1 |
|  | 5 | 4 | 3 | 2 | 1 |
|  | 5 | 4 | 3 | 2 | 1 |
|  | 5 | 4 | 3 | 2 | 1 |
|  | 5 | 4 | 3 | 2 | 1 |
|  | 5 | 4 | 3 | 2 | 1 |
|  | 5 | 4 | 3 | 2 | 1 |
|  | 5 | 4 | 3 | 2 | 1 |
